# Chronic pain and disability in organizations: It’s time to pay attention to work and workers

**DOI:** 10.1080/24740527.2021.2010023

**Published:** 2022-06-03

**Authors:** Duygu Gulseren

**Affiliations:** School of Human Resources Management, York University, Toronto, Ontario, Canada

The Canadian pain community knows that one in every five adults in Canada suffers from pain. What is less well known is that many of those adults stay in the workforce despite their pain. According to the Canadian Survey on Disabilities, more than half of working-age adults (i.e., between the ages 15 and 64 years) in pain are employed.^1^ Depending on the requirements of their jobs, these individuals continue to go to work, sit or stand on their feet during long shifts, work in awkward positions and in noisy environments, or lift heavy objects. Many also have comorbidities associated with pain such as anxiety or depression, which can make it harder for them to deal with difficult customers, travel for work, or stay focused on a task. In this editorial, I will (1) present a brief overview of the current state of the chronic pain and work literature, (2) make a case for why we need more pain-related research focused on the workplace and on employees with chronic pain who opt to continue working, and (3) invite pain researchers to collaborate with management scholars to better understand, prevent, and design interventions for chronic pain in the workplace.

Chronic pain is a prevalent problem in Canadian workplaces. The prevalence of pain in the workplace is expected to be even more common in the future because work conditions are changing. Factors such as an aging workforce, increasingly demanding jobs, longer work hours, and working more than one job are expected to cause an increase in chronic pain cases.

Despite the prevalence of pain in the workplace, very little research has examined the experiences of employees with pain. Although initial attempts have been made on this topic, significant and worrisome gaps exist in the literature. Except for general pain management interventions tested in workplace settings, most studies at the intersection of work and pain focus on absenteeism or on facilitating a return to work.^2^ Though these topics are important for our field, they assume, erroneously, that employees who have pain leave work and employees who return to work have no pain or pain that is effectively controlled; unfortunately, this is not the case for many. Employees who are working despite their pain are generally overlooked.

We need more research on this topic because the majority of the people with chronic pain are still in the workforce. They struggle with unique challenges at the intersection of the organizational life and their health conditions. Understanding what conditions at work help or hinder their pain and functioning could lead to the development of more effective interventions and policies.

Most individuals spend about one-third of their lives at work. Nonetheless, the influence of work is not limited to work hours and should not be underestimated. Work is a place where a person can earn their living, make social connections, and even find a sense of identity and purpose. Due to its wide sphere of influence in a person’s life, work has the potential to affect all biological, psychological, and social components that are represented in the biopsychosocial model of pain.^3^ This power, however, can be a double-edged sword: for some individuals work may be the source of their pain, whereas for others, work may mean access to pain management resources. Below, I will try to unpack this complex and intertwined relationship.

- **Work can cause pain**: It is no secret that poor working conditions lead to occupational conditions, including chronic pain. Numerous work-related factors such as performing physically demanding jobs, working for long hours, or being exposed to psychosocial hazards can all lead to the development of painful conditions.^4–7^ These factors can also trigger painful episodes for workers who already have a history of chronic pain.
- **Work can help manage pain**: Work means income for many workers. For employees in pain, it may also mean health benefits or insurance packages. In many parts of the world, including Canada, accessing health benefits through employment is only possible for full-time employees. This condition may make staying in the workforce a necessity for some employees for them to receive treatment for their pain.

Besides more tangible benefits such as compensation and benefits, employees may derive psychological and social benefits from work. For example, in a recent study we conducted,^8^ 13 full-time employees who had severe chronic pain reported using their work as a distraction from pain. Workers can make meaningful social connections at work, feel a sense of worth, and find purpose in life through their work.^8–11^

- **Employees with pain can be protected at work**: Chronic pain is a leading source of work disability in organizations. Depending on their individual condition or situation, employees in pain may leave the workforce. However, for those who opt to remain at work, many countries have laws that protect employees with disabilities. For example, in Canada, the Human Rights Act^12^ prohibits employers from dismissing employees based on their disability. Moreover, employers are required to provide reasonable accommodations to meet the work-related needs of their employees with disabilities. Employees with chronic pain are eligible for accommodation.

## A Call to Action

Uncovering the complex relationship between work and pain requires intimate knowledge of both organizations and pain, yet these two groups rarely cross paths in research settings. I believe meaningful progress in this area is only possible with the collaboration of these two parties. Organizational researchers can share insights on how workplaces are organized and jobs are designed; pain researchers can contribute expertise on how pain is assessed, managed, and prevented.

Such collaborations can enable understanding of the experiences of employees with pain better, improving the design of their jobs, developing more efficient interventions, and making policies that make a change for this group. Serving the needs of employees with chronic pain would also reduce the productivity and financial concerns of employers. Overall, these positive changes would improve the betterment of public health.

We can make meaningful contributions to understanding and improving the work lives of people with chronic pain conditions through research. These contributions can be even more impactful through collaboration with organizational researchers via interdisciplinary or intradisciplinary research. Canadian pain researchers have an international reputation for excellence given their pioneering role in advancing the field of pain research and management. Because of the multidisciplinary readership of the Canadian Pain Society’s flagship publication, I cannot think of a better place to share my invitation for such collaborations than the *Canadian Journal of Pain*.
